# Liquid Biopsy and Therapeutic Targets: Present and Future Issues in Thoracic Oncology

**DOI:** 10.3390/cancers9110154

**Published:** 2017-11-10

**Authors:** Paul Hofman

**Affiliations:** 1 Laboratory of Clinical and Experimental Pathology, Pasteur Hospital, 30 Avenue de la Voie Romaine, 06001 Nice, CEDEX 01, France; hofman.p@chu-nice.fr; Tel.: +33-4-9203-8855; Fax: +33-4-9203-8750; 2 Côte d’Azur University, FHU OncoAge, 30 Avenue de la Voie Romaine, 06001 Nice, CEDEX 01, France; 3 Hospital-Integrated Biobank (BB-0033-00025), Pasteur Hospital, 30 Avenue de la Voie Romaine, 06001 Nice Cedex 01, France

**Keywords:** liquid biopsy, lung cancer, *EGFR*, *ALK*, stratified medicine

## Abstract

The practice of liquid biopsy (LB) has revolutionized the care of patients with metastatic lung cancer. Many oncologists now use this approach in daily practice, applying precise procedures for the detection of activating or resistance mutations in *EGFR*. These tests are performed with plasma DNA and have been approved as companion diagnostic test for patients treated with tyrosine kinase inhibitors. *ALK* is another important target in lung cancer since it leads to treatment of patients who are positive for a rearrangement in *ALK* identified with tumor tissue. By analogy with *EGFR*, LB for detection of genomic alterations in *ALK* (rearrangements or mutations) has been rapidly adopted in the clinic. However, this promising approach has some limitations and has not yet been disseminated as much as the blood test targeting *EGFR*. In addition to these two therapeutic targets LB can be used for evaluation of the genomic status of other genes of interest of patients with lung cancer (*ROS1*, *RET*, *NTRK MET*, *BRAF*, *HER2*, etc.). LB can be performed to evaluate a specific target or for a more or less complex panel of genes. Considering the number of potential targets for clinical trials, techniques of next-generation sequencing of circulating DNA are on the rise. This review will provide an update on the contribution of LB to care of patients with metastatic lung cancer, including the present limits of this approach, and will consider certain perspectives.

## 1. Introduction

The term “liquid biopsy” (LB) has been created by comparison of the preexisting term tissue biopsy. In fact, this term LB may be replaced one day by a term such as, a “tumor targeted blood test”, which is more comprehensible to patients and the general public. So, the word “biopsy” (from Greek βίος, bíos (“life”) and Öψις, ópsis (“vision”)) can lead to confusion and the term “liquid biopsy” should, strictly speaking, be reserved for blood sampling and morphological analysis by microscopy of tumor cells isolated from blood. Thus, this term should not be used for methods of detection of circulating nucleic acids associated with molecular biology analyses, but only for the detection of circulating tumor cells (CTCs). However, for the sake of simplification, this term encompasses all techniques and is now associated with various objectives (screening and diagnosis of solid tumors and prediction of response to targeted therapy) [1].

Routine LB emerged rapidly in the clinic for the care of patients with cancer [2,3,4,5]. However, it is important to distinguish between LB used for clinical research projects and development and LB performed within the framework of healthcare providing immediate benefit to the patient, in particular for those with lung cancer. In this context several therapeutic targets can be detected with circulating nucleic acids (Table 1). This review will provide details concerning the potential of LB and its theranostic application for patients with non-small cell lung cancer (NSCLC) and will discuss the limits and perspectives of this domain.

## 2. Mutations in EGFR: The Main Therapeutic Target Examined in Daily Practice Using a Liquid Biopsy from Patients with Metastatic Non-Small Cell Lung Cancer

Activating and resistance mutations in *EGFR* can be detected with tissue biopsies or on cytological tumor samples from patients with metastatic NSCLC. These patients may benefit from targeted therapy with tyrosine kinase inhibitors (TKI). The same mutations can be detected with blood circulating tumor DNA extracted from plasma of patients [6]. This approach is now approved for treatment with TKI when a metastatic cancer is found and when it is impossible to obtain DNA from tissues or cells (fragile patients for whom sampling cannot be made, insufficient amount or quality of the tumor DNA) [7,18]. In this context, LB is a very useful tool that can be largely deployed in many hospitals for care of lung cancer patients, in particular when no tissue biopsy sample is available for these patients [8]. However, it is during the phases of tumor progression or relapse on treatment with TKI that LB is even more useful to detect resistance mutations in *EGFR*, in particular the T790M mutation that is sensitive to third generation TKI [9]. International recommendations advise first performing a LB in the case of progression in patients under these TKI treatments. If this resistance mutation is found after initial detection of the activating mutation, the patient is treated with the new generation of TKI. When no resistance mutation is detected in association with an activating mutation, a tissue biopsy must then be performed to look for a resistance mutation or other mechanisms that may be associated with this resistance [10,11]. When an activating mutation is initially detected with plasma DNA in the absence of an associated resistance mutation, a tissue biopsy is also recommended to look for an *EGFR* resistance mutation or other mechanisms of resistance, even if some of the latter can be detected with a LB (Table 2). Finally, when only one resistance mutation is found caution is necessary and the result must be confirmed with another method [18]. Overall, the sensitivity for detection of a mutation in *EGFR* in blood compared to tissue is estimated at between 60% and 70%. Several techniques with variable sensitivities are now available [12]. The two approaches approved in the USA by the Food and Drug Administration (FDA) are the COBAS (Roche Diagnostics) and the Therascreen (Qiagen) methods. A number of very sensitive methods such as digital PCR and new sequencing methods hold promise but need to be validated by each laboratory before routine use [13,14,15,16,38,39]. It is noteworthy that the diagnosis of the origin of some metastases occurring from an initially unknown lung cancer and finally from an adenocarcinoma of the lung can be made exceptionally in the absence of any tissue biopsy examination, if an *EGFR* mutation is detected with circulating DNA extracted from plasma [40]. Different mechanisms of secondary resistance can occur in patients treated with osimertinib and may be detected with a LB, such as the emergence of a small tumor cell subpopulation carrying the *EGFR*-C797S mutation, an additional subpopulation carrying amplified copies of *EGFR*-exon19del or other genomic alterations [10,41]. The different mechanisms of resistance occurring in patients receiving osimertinib and detected with a LB can be observed in patients receiving this TKI as second but also as first-line treatment of *EGFR* mutation positive [17,42,43]. Even if in the large majority of cases, the *EGFR* mutations are detected in an automatized manner from DNA extracted from plasma, these mutations have also been found in CTCs [44,45]. However, currently, despite numerous promises that have emerged from this specific domain of LB, this application is not used in a daily routine practice for *EGFR* mutational assessment and no automatized test has been approved to date for this by the FDA [46]. Different reasons can explain this limited interest in using CTCs as a possible target for determination of the *EGFR* mutation status: the difficulty of using a sensitive and specific method for CTC detection, the small number of CTCs in blood samples, and the phenotypic variabilities of CTCs, in particular due to the epithelio-mesenchyma transition phenomena [46,47].

## 3. Evaluation of the *ALK* Status with a Liquid Biopsy for Metastatic NSCLC

As for *EGFR* the detection of an *ALK* rearrangement can be done with a LB, at the time of diagnosis of the disease, when a tissue biopsy cannot be performed or when the RNA from tissue sample is quantitatively or qualitatively inadequate [19,20,21,22,25]. Several targeted methods can be used, including RT-PCR with plasma RNA or a platelet extract, multiplex analysis of a limited number of genes looking for fusions in *ALK* as well as in *ROS1*, *RET* and/or *NTRK* or analysis of an extensive panel of a large number of genes using next-generation sequencing (NGS) approaches [19,20,21,22,25]. Regardless of the approach used, the sensitivity of the tests is globally lower than for the reference tests performed with tissue to evaluate the *ALK* status (e.g., immunohistochemistry or Fluorescent *in situ* Hybridization (FISH) [19]. Several factors lead to this lower sensitivity, in particular the difficulty in mastering the pre-analytical steps after blood sampling, which result in rapid degradation of circulating plasma RNA. Aside from this, the amount of circulating tumor RNA can be insufficient depending on the sensitivity of the technique [19]. The *ALK* status can also be evaluated using circulating tumor cells (CTCs). Thus, several studies show the feasibility of detection by immunocytochemistry and/or FISH of the expression, rearrangement and/or amplification of *ALK* [23,24,50]. These studies show both good correlation between the results obtained with liquid and tissue biopsies and the potential of using CTCs for detection of *ALK* status in response to specific inhibitors of *ALK* [23,24,50].

It is well established that patients treated with inhibitors targeting the *ALK* rearrangement develop resistance, in the more or less short-term, in particular due to the emergence of *ALK* mutations [25,48]. These mutations can be detected with DNA from either tissue or plasma [19,25]. Several panels of genes include a more or less exhaustive list of *ALK* mutations [19,25]. In this context LB allows regular monitoring of patients treated with *ALK* inhibitors and therefore detection of mutations in blood at a very early stage with subsequent consequential modification of the therapeutic approach in the case of progression or relapse of the tumor [25]. Additionally, other mechanisms of resistance can be detected with a tissue biopsy and also with a LB (Table 2).

## 4. What Is the Future for Targeted Research into Activating Genomic Alterations on Genes Other Than EGFR or ALK When Using a Liquid Biopsy in NSCLC?

In theory, analyses with allele specific PCR, RT-PCR, digital PCR, FISH or immunocytochemistry can, depending on the genomic alteration concerned, be used to detect genomic alterations in genes other than *EGFR* or *ALK* (Table 2). In practice these approaches are not yet used routinely for patients with metastatic NSCLC. A number of examples are cited below.

### 4.1. Rearrangements in ROS1, NTRK, and RET

As for the *ALK* gene, rearrangements in *ROS1*, *NTRK* or *RET* can be detected with a LB and extraction of plasma RNA or CTC analysis, even if these detections are not done routinely to date as it can be done from tissue biopsies [26,27,28,29,30]. The constraints and limits of these approaches are similar to those mentioned above for *ALK*. Dedicated panels have been set up to analyze these rearrangements with LB, but optimization of the sensitivity of these techniques is needed to improve care of patients in routine practice. Evaluation of the *ROS1* status using FISH analysis with CTCs can also be envisaged, but it seems that this approach is difficult to implement immediately taken the constraints of the method [26,27]. Moreover, LB may allow to detect *ROS1* mutations occurring in patients treated with crizotinib [49].

### 4.2. Mutations in BRAF

The mutation *BRAFV600E* can be activated by certain drugs notably for patients with different solid tumors and *BRAF* mutations are present in 2–3% of lung adenocarcinomas [33]. The *BRAFV600E* mutation can be detected in lung cancer tissue by molecular biology techniques or with immunohistochemistry (IHC) [33]. This mutation can also be detected with LB by using different techniques [32,51].

### 4.3. Mutations in the RET and HER2 Genes

Targeted detection of *RET* or *HER2* is possible but has not, or minimally, been developed in practice using a LB approach in comparison with what is done from tissue biopsy to date [17,31,34,35].

### 4.4. Evaluation of the MET Status with a Liquid Biopsy

Detection of mutations in *MET* can lead to treatment of metastatic NSCLC [37]. Exhaustive analysis for these mutations is difficult since a number of panels do not presently cover all these mutations. Amplification in *MET* is one of the causes of secondary resistance to TKI that target activating mutations in *EGFR* and can be detected with a FISH analysis, but also by approaches such as next-generation sequencing (NGS). Thus, in theory, in a targeted manner, amplification in *MET* can be investigated by FISH analysis on CTCs from patients who progress on TKI targeting an activating mutation in *EGRF*, but no study has been performed to date on this aspect. Over-expression of the protein MET on CTCs has been reported for some patients with NSCLC and good correlation with the expression of MET in tissue biopsies performed on the same patients was recently observed [36].

## 5. Next-Generation Sequencing Approaches

Next-Generation Sequencing (NGS)approaches can be developed from circulating free-DNA (cf-DNA) and are now used by many teams to detect various genomic alterations with the same technology [22,52,53,54]. Several panels dedicated to a list of genes using NGS techniques have been commercialized specifically for studies with circulating nucleic acids [55,56,57]. In principle, these approaches can detect, depending on the case, mutations, rearrangements and/or amplifications. It remains to be seen if the combination of these approaches gives sufficient sensitivity and sometimes specificity, and if they are not affected too much by the pre-analytical phase for routine application, including the volume of blood samples used for DNA extraction [54,58,59].

## 6. Liquid Biopsy and Immuno-Oncology

Discussions around the use of LB within the context of immuno-oncology are recently ongoing [60,61]. In routine practice, the only predictive biomarker for therapeutic response to checkpoints inhibitors targeting the PD1/PD-L1 axis is PD-L1 when analyzed with immunohistochemistry (IHC). However, this IHC biomarker holds a number of weaknesses and other biomarkers must be rapidly considered, either in a combined fashion or as an alternative to IHC for PD-L1 assessment [62]. So, there is an urgent need to evaluate and to develop the use of LB to check if it can be a good tool to obtain a predictive value of the immunotherapy response (Table 3). In this context different approaches are under assessment and detailed below.

### 6.1. Analysis of Circulating Nucleic Acids

The tumor mutation burden (TMB) which is evaluated from tissue DNA with a targeted panel of certain genes seems to correlate with response to immunotherapy [74]. Thus, above and beyond PD-L1 IHC, integration of this parameter would allow distinction between patients with a good response with more than 50% of tumor cells expressing PD-L1 and patients with a low response but with also more than 50% of tumor cells expressing PD-L1. It is noteworthy that the TMB can be evaluated with circulating tumor DNA. In this context, recent studies have investigated the association between hypermutated circulating tumor DNA and the checkpoint inhibitor response [63]. Interestingly, a significant improvement in progression free survival was found to be associated with a high versus low alteration number in variants of unknown significance [63]. In the same way, and as envisaged for tumor tissue, it will be possible to evaluate the burden in neoantigens on circulating DNA, which has been found to be a biomarker for response to immunotherapy. The amount of circulating DNA could also correlate with the quality of the response to immunotherapy. In fact, a recent study showed that a decrease in the amount of circulating DNA was an indicator of good response and that this biomarker could participate in monitoring patients on immunotherapy [64]. Another possibility is to use LB as a predictive tool for an immunotherapy response by looking for chromosomal instability, which can be quantified by NGS from plasma/serum-derived cf-DNA [65]. In this regard it is possible to assess the predictive value of the chromosomal number instability (CNI) detected with cf-DNA as an early indicator of response to immunotherapy. Moreover, it has been demonstrated that the CNI score may distinguish cases of pseudo-tumor progression from cases of hyperprogression [65].

### 6.2. Analysis with Serum

Quantification of PD-L1 and PD-1 in serum is possible with notably the ELISA technique. Thus, some studies have shown that the amount of circulating PD-1 could represent a prognostic or theranostic biomarker [66]. However, certain pitfalls need to be considered, such as the fact that a number of inflammatory diseases can be associated with production of blood PD-L1/PD-1 and/or that the techniques have not yet been validated by studies performed with large cohorts of patients or in multi-center analyses. Finally, a recent study showed that quantification of interleukin 8 (IL-8) in serum of patients on immunotherapy may be of interest and that a drop in the level of IL-8 determined before treatment may be a biomarker of good therapeutic response [67].

### 6.3. Analysis of the Expression of PD-L1 on Circulating Tumor Cells

Several recent studies report the detection of PD-l1 on CTCs and the prognostic impact of this biomarker and even a correlation with the expression of tissue PD-L1. These studies concern different solid tumors such as breast, head and neck, bladder and lung carcinomas [68,69,70,71,72,73,75]. In particular PD-L1 expression can be detected in CTCs in NSCLC patients [72,73]. In one study, two distinct populations of nucleated, non hematolymphoid, PD-L1-expressing cells were identified; cytokeratin positive (CK+, PD-L1+, CD45−) and cytokeratin negative (CK−, PD-L1+, CD45) cells, both with cytomorphological features consistent of malignant tumor cells [70]. In this latter study, patients with a high count of PD-L1 positive CTCs had a worse overall survival than patients with a low count of PD-L1 positive tumor cells [70].

All these analyses used different direct and indirect methods of detection and characterization of CTCs and their validation with large cohorts of patients and multi-center projects is urgently necessary before envisaging implementation on a routine basis.

## 7. Conclusions

LB has suddenly become part of the daily routine of thoracic oncology, but its diffusion in hospitals and in the world is happening quite progressively [2,76,77]. The organization of healthcare system is heterogeneous from city to city, country to country and center to center and the set-up of circuits for sampling occurs at different levels depending on the individual organization. Mastering the pre-analytical phase is a major issue that directly impacts on the reliability of the results. In particular, the buffers used, the delay in transport of samples, the volume of the blood sample, the speed of centrifuge steps and the procedure for extraction of nucleic acids are all parameters for consideration when analyzing the results [78,79]. The sensitivity of the approaches varies and these techniques also influence the delay in transmission of the results. The latter being provided to the clinician a few hours or several days after blood sampling. It is noteworthy that only the search for *EGFR* mutations on circulating plasma DNA is presently based on standardized practices and on international recommendations, and authorizes administration of TKI. The other approaches and indications must be considered with strong precaution and skill, and the therapeutic decisions made in the interest of patients require multidisciplinary concretion and assessment. The different recent studies related to the LB field, as well as the continued optimization of the technologies, open new avenues and some very promising applications, particularly in thoracic oncology [80,81,82]. The hope that LB generated must also be reframed by certain limits and their use should be managed in an optimal way by oncologists [55]. The application of immunotherapy as an alternative to targeted therapy will certainly increase the indications for LB in the future. In this context, the latter could represent predictive biomarkers of these new treatments and participate in the monitoring proposed to patients.

The fields of investigation using LB are still relatively narrow in routine practice, in particular due to the number of unknowns, and promising techniques have not yet been sufficiently validated for transfer to the clinic [83,84]. The main interest in routine practice is to consider LB as an option for the identification and monitoring of *EGFR* mutations in late stage NSCLC, given the good rates of concordance with matched tissue biopsies when robust mutation testing tools are used. LB NGS approaches are needed to provide information identical to those obtained from tissue sample. Even if new technologies are under development, the sensitivity is probably not high enough to substitute tissue-based NGS approaches. Novel applications in thoracic oncology will probably emerge [85,86,87,88,89,90,91]. Several areas of investigation have been made on analyses of other blood components such as circulating exosomes and microRNAs [92,93,94]. These other investigations could open avenues for the discovery of new biomarkers which will be enough sensitive and specific to be use in clinical practice in the next future [92,93,94]. Finally, a degree of confusion still exists concerning the tools for detection and the results obtained for the detection and characterization of CTCs in thoracic oncology. This latter specific domain of LB is not yet adapted to daily use by pulmonologists and oncologists. While very promising, this field of application, in contrast to detection of mutations on *EGFR* with plasmatic DNA, still requires evaluation in comparative studies and validation by multi-centers [84,95,96,97,98].

## Figures and Tables

**Table 1:** Main genes and genomic alterations which can be detected in late stage NSCLC using tissue and/or liquid biopsies. +: worse approach; ++: intermediate option; +++: best approach.

Gene/Genomic Alteration	Detection by Liquid Biopsy [Ref]	Detection by Tissue Biopsy [Ref]
*EGFR*/mutations	+++ [6,7,8,9,10,11,12,13,14,15,16,17]	+++ [7,10,17,18]
*ALK*/rearrangement	+ [19,20,21,22,23,24]	+++ [22,25]
*ROS1*/rearrangement	+ [26,27]	+++ [27]
*NTRK*/rearrangement	+ [28]	+++ [29]
*RET*/rearrangement or mutations	+ [30]	+++ [31]
*BRAF*/mutations	+++ [32]	+++ [33]
*HER*2/mutations or amplification	++ [17]	+++ [34,35]
*MET*/mutations or amplification or expression	++ [17,36]	+++ [36,37]

**Table 2:** Main genomic alterations associating targeted therapies and mechanisms of resistance and detection efficiency using tissue and/or liquid biopsies in late stage non-small cell lung carcinoma. SCLC: small-cell lung carcinoma; EMT: epithelial to mesenchymal transition. +: worse approach; ++: intermediate option; +++: best approach.

Gene/Genomic Alteration	Example of Targeted Therapy	Main Mechanisms of Resistance [Ref]	Detection by Liquid Biopsy	Detection by Tissue Biopsy
*EGFR*/activating mutations	First- and second-generation *EGFR* TKIs	*EGFR* mutations [9,10,11,41]:	+++	+++
		T790M; A761T; T854A; L7981; L692V; E709K; L718Q, etc.		
		Alternative pathway activation [11]:	++	+++
		*MET* amplification; *HER2* amplification; *BRAF* mutation; *PIK3CA* mutation; *AXL* activation		
		Autocrine HGF production		
		Phenotypic transformation [11]:	(−)	+++
		SCLC; EMT		
*EGF*R/T790M resistance mutation	Third-generation *EGFR* TKIs	*EGFR* mutations [9,17,41,42,43]:	+++	+++
		C797S; C797G; G724S, etc.		
		*A*mplification [17,41,43]	+	+++
		*EGFR*; *HER2*; *MET*; *FGFR*1		
		T790M mutation disappearance [43]	+++	+++
		Activation [17,43]	(−)	+
		*IGFR1*		
		Phenotypic transformation [17,43]	(−)	+++
		SCLC; EMT		
*ALK*/rearrangement	Crizotinib Ceritinib Alectinib Lorlatinib Other	Specific *ALK* mutations [19,25,48]:	+++	+++
		L1196M; G1202R, F1174C; I1171T/N/S, etc.		
		*ALK* CNG [25]	++	+++
		Mutation [25]*:*	++	+++
		*PIK3CA*		
		Molecule activation [25,48]:	+	+++
		SRC; IGF-1R; Ligand-mediated HER2/3; Protein kinase C; MAPK pathway activation; MET amplification		
		Phenotypic transformation [25]:	(−)	+++
		SCLC; EMT		
*ROS1*/rearrangement	Crizotinib Ceritinib Lorlatinib Other	Specific *ROS1* mutations [49]:	++	++
		G2032R; G2026R; L2026M, etc.		

**Table 3:** Main developments for use of a liquid biopsy in the field of immunotherapy.

Blood Bioresource	Analysis [Ref]	Potential Interest
Circulating free DNA	Tumor mutation burden [63]	Predictive of response
	Quantification of DNA [64]	Predictive of response
	Chromosomal number instability [65]	Predictive of response
Serum	Circulating PD-1 [66]	Prognosis
	Interleukine-8 [67]	Predictive of response
Circulating tumor cells	PD-L1 expression on CTCs detected with an indirect method [68,69,70,71,72]	Prognosis and predictive
	PD-L1 expression on CTCs detected with a direct method [70,73]	Prognosis and predictive
